# Template subtraction based methods for non-invasive fetal electrocardiography extraction

**DOI:** 10.1038/s41598-024-51213-5

**Published:** 2024-01-05

**Authors:** Rene Jaros, Eva Tomicova, Radek Martinek

**Affiliations:** Department of Cybernetics and Biomedical Engineering, Faculty of Electrical Engineering and Computer Science, VSB-Technical University of Ostrava, 17. listopadu 2172/15, 708 00 Ostrava, Czechia

**Keywords:** Data processing, Biomedical engineering

## Abstract

Assessment of fetal heart rate (fHR) through non-invasive fetal electrocardiogram (fECG) is challenging task. This study compares the performance of five template subtraction (TS) methods on Labor (12 5-min recordings) and Pregnancy datasets (10 20-min recordings). The methods include TS without adaptation, TS using singular value decomposition (TS_SVD_), TS using linear prediction (TS_LP_), TS using scaling factor (TS_SF_), and sequential analysis (SA). The influence of the chosen maternal wavelet for the continuous wavelet transform (CWT) detector is also compared. The F1 score was used to measure performance. Each recording in both datasets consisted of four signals, resulting in a total comparison of 88 signals for the TS-based methods. The study reported the following results: F1 = 95.71% with TS, F1 = 95.93% with TS_SVD_, F1 = 95.30% with TS_LP_, F1 = 95.82% with TS_SF_, and F1 = 95.99% with SA. The study identified gaus3 as the suitable maternal wavelet for fetal R-peak detection using the CWT detector. Furthermore, the study classified signals from the tested datasets into categories of high, medium, and low quality, providing valuable insights for subsequent fECG signal extraction. This research contributes to advancing the understanding of non-invasive fECG signal processing and lays the groundwork for improving fetal monitoring in clinical settings.

## Introduction

Fetal heart rate (fHR) analysis is very important during pregnancy because it provides critical information about fetal health such as presence of fetal hypoxia. Currently cardiotocography (CTG) is so called golden standard for fetal monitoring. CTG was the first method used for fetal heart activity monitoring and helped to reduce number of fetal mortality due to mentioned hypoxia^1^. However, since CTG started being commonly used, the number of caesarean sections performed for presumed hypoxia has increased^2^. For this reason and also because of ultrasound energy, alternative methods started to be tested such as fetal electrocardiography (fECG)^3–5^, fetal phonocardiography (fPCG)^6–8^ and fetal magnetocardiography (fMCG)^9–11^. Analysis of the fECG is a useful for fHR detection and many adverse factors during pregnancy and childbirth. There is invasive and non-invasive approach to measure fECG. Invasive approach is dangerous and able to perform only during labour. Therefore, it is preferable to measure fECG by non-invasive approach using electrodes placed on maternal abdomen. The signal obtained by this way is called abdominal ECG (aECG) and typically contains a large amount of noise^1,12^.

Overall, noninvasive fECG extraction is a challenging task that is related to several factors associated with the physiology and technical aspects of signal acquisition and processing. The main problem is the low signal-to-noise ratio (SNR) of fECG component. Compared to maternal signals, these fetal signals are usually weak in aECG signals, which complicates the extraction of a clear fetal signal. In addition, there may be overlap between mECG and fECG signals, leading to contamination and difficulty in distinguishing between the two signals.

Another problem is the correct placement of the electrodes, which is crucial for obtaining aECG signals containing a sufficiently distinct fetal component (there is no standardised distribution). In general, we are talking about the quality of the input aECG signal. This can be difficult due to the small size of the fetus, its position and location. Fetal and maternal movements can also cause artifacts, making it difficult to obtain a stable and reliable signal. It is important to note that fECG signals change with gestational age, so most extraction algorithms need to adjust the input parameters to these changes. All these problems lead to difficulties in automatic determining the quality of the input aECG signal for subsequent processing (in case it is necessary to select a certain number of measured input signals to be processed).

Using multiple electrodes and processing signals from different locations can improve signal quality, but also introduces problems related to spatial resolution. Moreover, sometimes it is preferable to process only one input aECG signal (for example fECG signal extraction via mobile device). To make the processing of fECG signals more accurate, standardized protocols for data acquisition, signal processing and validation metrics need to be developed. However, due to the above problems, this is still an unsolved problem.

Currently, the aim is to test the applicability of fECG for home fetal monitoring. If all the issues could be debugged and HW and SW developed to accurately extract critical information non-invasive fECG could be used in clinical practice. This would mean a more accurate determination of fetal hypoxia compared to CTG, as the fECG signal allows determination of short-term changes in fHR. This means that sometimes there are accelerations in the fHR signal that are physiological, but in the case of CTG, they can be evaluated as a possible problem. In addition, using an accurately extracted fECG, it is possible to perform ST analysis, which can even more accurately determine possible hypoxia. Which could also lead to the replacement of the ST analyzer.

There are already several commercially available devices for measuring non-invasive fECG. These are mainly the Monica AN24 (2012) and Monica Novii Wireless Patch System (2014) (Monica Healthcare Ltd., Nottingham, UK)^13^, MERIDIAN M110 Fetal Monitoring System (2017) (MindChild Medical, Inc., North Andover, MA, USA)^14^, and PUREtrace (2017) (Nemo Healthcare, Veldhoven, the Netherlands)^15^.

Currently, there are a large number of extraction methods. Researchers are still struggling with this problem and try to find a method to reliably and efficiently extract fECG^16–22^. The extraction approach can be divided into single-channel and multi-channel with each type of method having its own advantages and disadvantages. The most commonly used methods for fECG extraction are multi-channel and include methods based on blind source separation (BSS) such as independent component analysis (ICA) and Principal component analysis (PCA)^1,23–25^. Furthermore, methods based on adaptive algorithms such as least mean squares (LMS) and recursive least squares (RLS) are also very commonly used^26–28^. However, as far as single-channel methods are concerned, template subtraction (TS) based methods seem to be suitable in terms of simplicity and efficiency. There is a simple variant of the TS method without any template adaptation, but in addition there are several variants based on different approaches to template adaptation.

TS is an fECG extraction method that better suppresses misaligned fetal R-peaks and re-estimates missing individual R-peaks, thereby trying to find overlapping intervals of mECG and fECG signal^17^. Authors in the study^29^ explored the use of singular value decomposition (TS_SVD_) to create a template. In study^30^ they focused on predicts an upcoming complex from previous by linear prediction TS_LP_. The authors in the^31^ study took a different approach to template creation by using a scaling factor TS_SF_. Sequential analysis (SA) is an extraction method using a priori information about the maternal R-peaks, where this information is used to detect the mECG signal and create a template by applying averaging and scaling, which leads to improved extraction success rates^32^. In the study^33^, the authors compared BSS methods, adaptive filters and also three different TS-based methods namely TS_SVD_, TS_SF_, and TS using Extended Kalman Filter (TS_EKF_). They came to the results that TS methods achieved an median F1 value of 96.0%, which was lower than using adaptive filtering (97.9%) and BSS methods (99.9%). However, it should be emphasized that they used synthetic data from the fECGSYNDB and discussed that a certain algorithm may work well in some particular cases and fail in some non-stationary cases. The use of different TS-based methods was also mentioned in the study^34^, which aimed to create a practical guide for noninvasive fECG signals processing. Other methods that use a template include for example Dynamic time wrapping (DTW). This method takes into account the diffeomorphism of each period by adjusting the subtraction template^12^. Template-matching (TM) approach is aimed at localizing the fECG R wave that overlapped with mQRS. This method uses several principal components from the multi-QRS subspace decomposition using SVD to construct the mQRS and fQRS templates^35^. Group of BSS methods are used to estimate or separate mECG from the sensor without knowing the characteristics of the transmission signal, where the ICA approach is very often used^36^. However, these methods require multichannel signal sources. The non-negative matrix (NMF) factorization method is used to separate fECG using activation scaling by scaling a specific row of the activation matrix, the signal of interest can be emphasized from the mixed signal^37^. The use of a time-frequency analysis combining the fractional Fourier transform (FrFT) and the discrete wavelet transform (DWT), called FrFT-DWT, for fECG extraction is discussed in the study^38^. Last but not least, the Stockwell transform (ST) method is used to represent the signal in the time-frequency domain. It is an extension of the short-time Fourier transform with a Gaussian window with scalable width. The identification of the maternal R-peaks uses a time-frequency domain mapping converted into a one-dimensional unipolar signal^39^.

In addition to the fECG extraction and signal filtering, R-peaks detection is a very important part of fHR estimation. This step is very critical because even with very accurate fECG extraction, the detection alone can invalidate the entire result. Currently, a detector based on the continuous wavelet transform (CWT) is considered as a very accurate^40,41^. However, many experiments show that the main influence on CWT detector efficiency is the correct choice of the maternal wavelet type.

Efficiency of TS based methods varies greatly depending on the input aECG signals, so in this paper we will focus not only on a comparison of TS based methods used for mECG elimination/reduction, but also on performing the experiment on different databases containing real recordings. As we mentioned, the final estimated fHR depends also on used R-peak detectors, so influence of maternal wavelet type on CWT detector accuracy will be performed. Main contribution of this study include:

- Comparative analysis of maternal wavelet used for CWT detector.
- Comparative analysis of TS based methods.
- Experiment on real datasets with reference annotations.
- Determination of suitable input aECG signals from each recording for fECG extraction.

The rest of the paper is organized as follows: “Material and methods” will provide state of the art about TS based methods. “Proposed methodology” will include the materials and methods used along with the methodology of the experiment conducted. “Results” will contain the results of the experiments along with the resulting method comparison. Discussion and conclusion will be presented in the “Discussion” and “Conclusion”.

## Material and methods

Extracting the fECG signal using TS methods has been shown many times to be very accurate. However, a comparison of the different TS-based methods has not yet been sufficiently performed to demonstrate which one is appropriate. A big problem is the test run on only one recording or one type of dataset, because the experiment result may turn out differently on another dataset. This means that the accuracy of extraction methods is strongly dependent on the input signals used. For this reason, two different datasets containing real signals were used in this study. Each recording of the used datasets contains four aECG signals, so we will also focus on testing the extraction result on each signal and determine suitable one. Furthermore, as already mentioned, the extraction result itself is strongly dependent on the detector used or its setup, therefore in this work an experiment was performed focusing on the selection of a suitable mother wavelet for a very efficient CTW detector.

### Dataset

We used signals from two real datasets available on a public server, and were recorded under clinical conditions as part of research projects at the Department of Obstetrics and Gynecology of the Medical University of Silesia in Katowice, Poland. Research was approved by the University’s Bioethics Committee (Commission approval number NN-013-345/02). The subjects read the informed consent and gave a written consent to participate in the study. The datasets analysed during the current study are available in the figshare repository integrated with Scientific Data Journal, detailed information could be found in Refs.^42,43^.

These datasets are consisting of four aECG signals that were obtained by non-invasive measurement (Ag/AgCl electrodes were placed on the maternal abdomen). All signals were recorded as part of research projects at the Department of Obstetrics and Gynecology of the Medical University of Silesia in Katowice, Poland. The recording of the signals was always supervised by qualified trained medical personnel^43^. Both datasets are annotated with the exact positions of the fQRS complexes, which were determined by the authors using automatic detection of R-peaks and verified by clinical experts.

**Figure 1:**
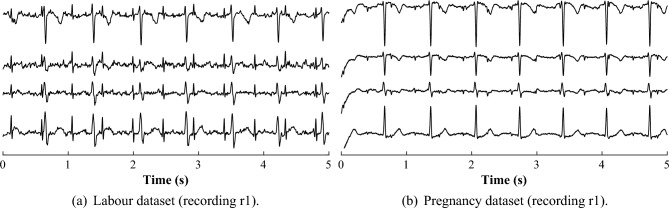
Sample aECG signals from the datasets used for the experiments.

The signals from both datasets were digitized with 16-bit resolution and a sampling rate of 500 Hz. All captured aECG signals were preprocessed using a filter with multiple notches located every 50 Hz. To eliminate low frequency interference, the cutoff frequency was set at 5 Hz and to eliminate power line interference, the cutoff frequencies were set between 45 and 55 Hz. Labor dataset contains 12 recordings of 5 min in length originating from women between 38th and 42nd week of pregnancy taken in an advanced stage of labor. Pregnancy dataset contains 10 recordings of 20 min in length originating from women between 32nd and 42nd weeks of pregnancy. Figure 1 shows samples of the r1 recordings from both datasets.

### Evaluation parameters

In this work, the objective evaluation is performed by calculating the accuracy of R-peaks detection. In order to compute the accuracy parameters, we first need to extract the fECG signal and estimate the R-peaks positions in it. Furthermore, the datasets under test need to have reference annotations of the correct R-peaks positions determined by experts. Subsequently, the parameters true positive (TP), false positive (FP) and false negative (FN) are determined. Detected R-peaks in the extracted signal that are within ± 50 ms of the reference annotations are marked as TP. FP is defined as detected R-peaks in the extracted signal that fall outside the mentioned interval. Finally, missed R-peaks that should have been detected in the mentioned interval but were missing are determined as FN. After determining these parameters TP, FP and FN, it is possible to calculate sensitivity (SE) using Eq. (1), calculate positive predictive value (PPV) using Eq. (2) and calculate F1 score using Eq. (3)^44–47^.

1 $$\begin{aligned} SE&= \frac{TP}{TP+FN} \cdot 100. \end{aligned}$$

2 $$\begin{aligned} PPV&= \frac{TP}{TP+FP} \cdot 100. \end{aligned}$$

3 $$\begin{aligned} F1&= 2 \cdot \frac{SE \cdot PPV}{SE+PPV}. \end{aligned}$$

### Proposed methodology

**Figure 2:**
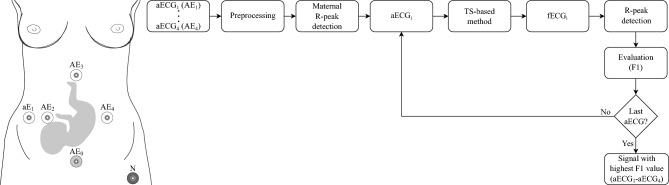
Flowchart of experiment used for fECG extraction (AE_1_–AE_4_ are active electrodes, AE_0_ is reference electrode, and N is active ground).

In this subsection, each significant parts of the experiment will be described in more detail. All methods were performed in accordance with the relevant guidelines and regulations. Figure 2 shows the procedure of the experiment conducted in this study. The demonstration is performed for a single recording from used datasets containing four input aECG signals. The whole experiment can be divided into several steps:

1. Preprocessing of input aECG signals (four signals are always used for a single recording from used datasets).
2. Detection of maternal R-peaks from input aECG signals (use of PCA, rules and CWT detector).
3. Input signal selection for further processing (processing sequentially all four input aECG signals measured by the electrodes AE_1_–AE_4_).
4. Extraction of fECG signal using TS method (using one type of TS-based method, because the experiment was always performed for each method separately).
5. Detection of fetal R-peaks from extracted fECG signal (labeled as fECG_i_ in the flowchart because it depends on the input aECG signal being processed) using CWT detector.
6. Evaluation by F1 score, which indicates the harmonic mean between sensitivity and positive predictive value, and storing the result.
7. Repeating steps 3–6 for the remaining aECG signals.
8. Determine the input aECG signal with the highest F1 value from the tested input aECG signals.

Thus, the experiment was performed repeatedly for each tested TS-based method for subsequent comparison of their performance. In the next subsection a description of the important parts of the presented experiment will be made: Preprocessing, Maternal R-peaks Detection, and Template Subtraction.

#### Preprocessing

Both technical and physiological interferences are present in aECG measurements. The physiological interface is associated with manifestations of the organism, such as motion artifacts (at high frequencies), breathing activity (at low frequencies) or signal interference from other biological signals. On the other hand, technical interference mainly includes power interference (50 Hz; 60 Hz). However, apart from these artifacts, the biggest problem (artifact) during fECG extraction is the maternal signal, which is several times larger in amplitude than the fetal signal. In addition, the spectrum of mECG overlaps with fECG, making fECG extraction more complicated. The main frequency of maternal QRS (mQRS) complexes lies in the range of 0.5–35 Hz and the main frequency of fetal QRS complexes lies in the range of 10–15 Hz^17,43,48,49^.

In this study for preprocessing we have chosen finite impulse response (FIR) filter. Since the data was bandstop filtered (45–55 Hz) and highpass filtered (5 Hz) by the dataset authors^50,51^, we only used the bandpass filter. Considering the aforementioned frequency band of fetal QRS complexes and dataset authors filtering, we used a band of 5–70 Hz and a filter order of 500.

#### Maternal R-peaks detection

TS-based methods require accurate determination of the maternal R-peak positions, because without this step it is not possible to create a template for adaptation and subsequent subtraction. This step is very important because if maternal R-peak positions are inaccurately determined, it introduces a large error into the extraction process itself. The datasets used have annotations regarding the exact maternal R-peak positions established by experts. However, we could not use them for our purposes because in practice, when measuring and analyzing signals, we do not have information about the exact positions of the maternal R-peak positions, so we need to determine them.

The algorithm for detecting and determining maternal R-peak positions is based on the following procedure. Since we always have four input aECG signals for our experiment, we can perform a more accurate maternal R-peaks detection. First, all aECG signals are used as input to PCA to find the main source signals and eliminate the problem of poor input selection where the detection would not be accurate. PCA is a dimensionality reduction technique and its primary goal is to transform a dataset with potentially correlated variables into a new set of uncorrelated variables, known as principal components. These components are linear combinations of the original variables and are ordered by the amount of variance they capture in the data^1^.

Subsequently, the CWT detector is used to detect R-peaks. The CWT detector is based on the decomposition of the signal by CWT to the 5th level. Subsequently, a search for local minima and maxima in the received signal after CWT is performed. Further, the adjustment of the searched local minima and maxima is performed using adaptive thresholding. Finally, zero-crossing detection is performed between the adjusted local minima and maxima that are separated by a maximum of 120 ms (modulus pair). The last modification is to find the maximum (R-peak) in the neighborhood of the detected zero passes^52–54^.

CWT detector is applied at the first and second outputs of the PCA method. This is because these two outputs have the highest energy and by using both of them, we avoid the problem of having only the fetal signal without the maternal component in the first estimated signal. Then, algorithm decides which of the PCA outputs provided the smaller number of R-peaks and this is selected as the correct one. The determined positions are stored and prepared for TS based methods.

We subsequently checked the accuracy of the maternal R-peaks detection against the reference annotations provided for the databases. We used the F1 score determination for the evaluation and achieved an accuracy of 99.75%, confirming that the proposed algorithm is sufficient for the purpose of this study. Minor inaccuracies are not caused by bad algorithm and rules, but by the quality of the input signals and the functionality of the CWT detector. Regarding the CWT detector settings, the maternal wavelet gaus1 was chosen.

#### Template subtraction

**Figure 3:**
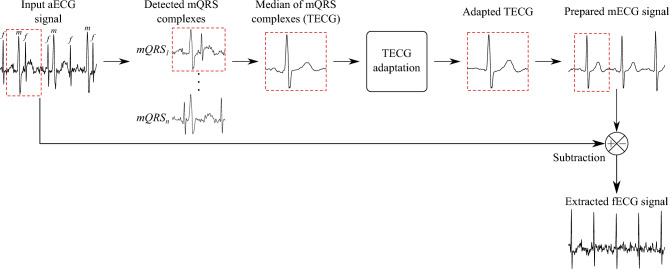
Block diagram of TS method functionality.

The TS method is simple and effective single-channel fECG extraction method. Figure 3 shows a diagram of the TS method functionality. At the beginning of the TS method, it is necessary to detect the positions of R-peaks in the input aECG signal. Then, based on these positions, individual mQRS complexes are cut out (0.25 s to the left and 0.45 s to the right of the determined R-peaks). Subsequently, a template is created by median of all received mQRS complexes^17^. Finally, a template subtraction is performed at all locations where maternal R-peaks were originally detected. This removes the maternal signal from the input aECG signal, leaving ideally only the fECG signal.

There are many variations of this method aimed at template adaptation. This means that unlike the classical TS method described above, which takes the template and subtracts it at the individual locations of the mQRS complexes, it additionally adapts the shape of the template to the actual mQRS complex to be subtracted. This will greatly increase the accuracy of the estimated fECG single. The selected template-based methods for this study are described below:

- *Template substraction using singular value decomposition* (TS_SVD_) SVD is a factorization of certain input matrix into a matrix *U*, $\Sigma$ and *V*, where *U* and *V* are orthonormal matrices and $\Sigma$ is a zero matrix except for possible non-negative numbers on the main diagonal (these numbers are called singular values of the input matrix). The disadvantage is that the computational complexity of constructing the singular decomposition increases with the third power of the dimension of the matrices. The TS_SVD_ method estimates matrix *U* from matrix of detected mQRS complexes with selected number of source components, see Eq. (4) for SVD calculation. This matrix *U* is then used to create the template *TECG* relative to the actual $mQRS_i$ complex from the input aECG signal to be subtracted, see Eq. (5)^29^.
  4 $$\begin{aligned} SVD&=U \cdot \Sigma \cdot V^T. \end{aligned}$$
  5 $$\begin{aligned} TECG&=mQRS_i \cdot (U \cdot U^T). \end{aligned}$$
- *Template substraction using linear prediction* (TS_LP_) This method uses linear prediction to determine the template (predicts an upcoming complex from previous). The template is constructed by weighting the previous cycles to minimize the root mean square error (unlike other TS-based methods where the weights of each cycle are the same). In order to adapt the *TECG* template to the actual $mQRS_i$ complex, this method uses the Eqs. (6) and (7), where $mQRS_i$ is the actual mQRS complex, *mQRS* is a matrix whose rows are the individual mQRS complexes and vector $\lambda$ are contains weights^30^.
  6 $$\begin{aligned} \lambda&=(mQRS^T \cdot mQRS)^{-1} \cdot mQRS^T \cdot mQRS_i. \end{aligned}$$
  7 $$\begin{aligned} TECG&=\lambda \cdot mQRS. \end{aligned}$$
- *Template substraction using scaling factor* (TS_SF_) This method is based on determining a scaling factor for template adaptation. After preparing the template using the median, Eq. (8) is used to calculate the scaling factor for the actual complex $mQRS_i$. Then, according to Eq. (9), the *TECG* template is adjusted to the actual complex $mQRS_i$ and used for subtraction. The scaling reduces the discrepancy between the average and true mQRS complex, which is affected by the time-varying morphology of mECG signal^31^.
  8 $$\begin{aligned} a&=(TECG^T \cdot TECG)^{-1} \cdot TECG^T \cdot mQRS_i. \end{aligned}$$
  9 $$\begin{aligned} TECG&=\alpha \cdot TECG. \end{aligned}$$
- *Sequential analysis (SA)* SA is based on TS_SF_ method and focused on scaling procedure improvement. Scaling is not performed on entire mQRS complexes, but separately scales the P wave, QRS complex and T wave. In this way, the temporal variability of the morphology of the mECG signal is considered. The prepared template *TECG* is divided into P wave (0–0.2 s of template), QRS complex (0.2–0.3 s of template) and T wave (0.3–0.7 s of template). Scaling factors $a_p$, $a_{QRS}$ and $a_T$ are then determined for each segment using Eq. (8), which are used to adapt the template (using Eq. (9)) before subtracting the actual $mQRS_i$ complex^32^.

## Results

The whole experiment on real data from the Labour and Pregnancy datasets was performed on all signals of each recording. The MATLAB R2023a programming language was used. In the first part of the results, the effect of the used maternal wavelet on the accuracy of R-peak detection was tested. All extracted signals were successively used as input to the CWT detector where the maternal wavelets were tested:

- *Biorthogonal:* bior1.1, bior1.3, bior1.5, bior2.2, bior2.4, bior2.6, bior2.8, bior3.1, bior3.3, bior3.5, bior3.7, bior3.9, bior4.4, bior5.5, bior6.8
- *Coiflet:* coif1–coif5.
- *Daubechies:* db1–db45.
- *Fejer-Korovkin:* fk4, fk6, fk8, fk14, fk18, fk22.
- *Gaussian:* gaus1–gaus8.
- *Reverse biorthogonal:* rbio1.1, rbio1.3, rbio1.5, rbio2.2, rbio2.4, rbio2.6, rbio2.8, rbio3.1, rbio3.3, rbio3.5, rbio3.7, rbio3.9, rbio4.4, rbio5.5, rbio6.8.
- *Symlet:* sym1–30.

A total of 124 different maternal wavelts were tested. To summarize the extraction efficiency, the mean of all F1 obtained on all signals of one dataset was then performed. Subsequently, the same was done for the second dataset. Finally, a similar test was performed for both datasets together. From the received table, which had 124 rows, only a part of the best results was selected. Other results can be found in the supporting material. These results can be seen in Table 1, where the highest value in a given column is highlighted in bold. From this table it can be deduced that the wavelets with a lower width index achieved better results than those with a higher index. This implies that narrower wavelets are preferable for R-peak detection. It can also be seen that the Gaussian family wavelets achieved the highest accuracy, with the gaus2 wavelet achieving the highest accuracy for the Labour dataset and then the gaus3 wavelet for the Pregnancy dataset. Moreover, the gaus3 wavelet achieved the highest accuracy when the experiment was performed on both datasets together.

**Table 1:** Effect of maternal wavelet selection for CWT detector on R-peak detection accuracy.

Maternal wavelet	F1 score (%)		
	Labour dataset	Pregnancy dataset	Both datasets
bior1.1	94.05	91.45	92.87
bior1.3	93.95	95.25	94.54
bior1.5	93.76	95.17	94.40
coif1	93.21	94.35	93.73
db1	94.05	91.45	92.87
db2	93.27	94.46	93.81
db3	92.31	92.41	92.35
db4	91.28	93.46	92.27
fk4	93.58	94.70	94.09
fk6	91.61	92.75	92.13
gaus1	94.72	95.80	95.21
gaus2	**95.21**	96.21	95.66
gaus3	94.80	**96.89**	**95.75**
gaus4	93.95	96.16	94.96
gaus5	92.80	94.50	93.58
rbio1.1	94.05	91.45	92.87
rbio2.2	94.13	95.66	94.82
rbio2.4	92.62	93.64	93.08
rbio3.1	94.62	95.09	94.84
rbio3.3	93.59	95.18	94.31
rbio3.5	92.67	94.16	93.34
rbio4.4	92.27	93.47	92.81
rbio5.5	91.71	92.49	92.07
sym1	94.17	91.45	92.93
sym2	93.34	94.46	93.85
sym3	92.35	92.44	92.39

Significant values are in bold.

Based on the initial experiment with the influence of the maternal wavelet, it was decided to use the gaus3 wavelet for the rest of the experiment. Tables 2 and 3 then show the results of the accuracy of R-peak detection based on the F1 determination on each dataset. Using each TS-based method, fECG signal extractions were performed on all signals of each recording of both datasets. For clarity, results greater than 90% (high) are highlighted in bold and results less than 80% (low) are highlighted in italic. The remaining results in the 80–90% interval have been left in black (medium). These results indicate which signals of each recording are applicable for fECG signal extraction, and also which TS-based method provides the highest accuracy.

**Table 2:** Results of the accuracy of R-peaks (F1) determination from extracted signals using individual tested TS-based methods on the Labour dataset (using the gaus3 maternal wavelet).

TS-based method	Channel	Recording											
		r1	r2	r3	r4	r5	r6	r7	r8	r9	r10	r11	r12
TS	aECG 1	**99.14**	**95.08**	*60.91*	**96.86**	**96.58**	**99.12**	*31.75*	**98.45**	**94.98**	*20.18*	**98.61**	**92.96**
	aECG 2	**98.29**	**92.25**	*56.94*	**93.96**	**96.13**	**98.10**	**94.39**	**97.83**	**94.39**	**95.06**	**95.38**	85.97
	aECG 3	**98.29**	*38.64*	*34.90*	*50.34*	**97.27**	*40.14*	88.75	**99.84**	*40.00*	**96.02**	**97.54**	**96.14**
	aECG 4	**99.45**	84.30	*59.29*	88.55	**98.33**	**96.65**	**95.57**	**100.00**	85.80	**100.00**	**97.38**	**99.85**
TS_SVD_	aECG 1	**99.30**	**95.08**	*59.92*	**96.79**	**97.34**	**99.12**	*31.80*	**96.98**	**93.59**	*21.52*	**98.61**	**92.96**
	aECG 2	**98.45**	**91.72**	*55.83*	**92.86**	**95.81**	**97.95**	**95.34**	**97.52**	**94.18**	**97.77**	**94.76**	86.69
	aECG 3	**98.45**	*37.58*	*35.08*	*53.46*	**95.68**	*42.06*	88.49	**99.69**	*38.76*	**96.25**	**98.46**	**95.77**
	aECG 4	**99.45**	83.93	*61.99*	**90.21**	**98.33**	**95.33**	**96.76**	**100.00**	**90.59**	**99.68**	**97.84**	**100.00**
TS_LP_	aECG 1	**98.99**	**94.38**	*60.91*	**96.72**	**95.14**	**98.39**	*31.75*	**98.06**	**94.47**	*20.39*	**97.61**	**92.81**
	aECG 2	**97.82**	**91.70**	*56.58*	**93.09**	**95.52**	**97.37**	**93.69**	**97.06**	**94.16**	**94.50**	**94.99**	85.99
	aECG 3	**98.14**	*38.57*	*34.68*	*49.96*	**96.13**	*41.54*	88.50	**99.30**	*39.95*	**95.45**	**97.07**	**95.46**
	aECG 4	**98.76**	84.45	*59.55*	88.03	**97.04**	**96.36**	**94.86**	**99.22**	85.53	**98.88**	**96.60**	**99.46**
TS_SF_	aECG 1	**98.76**	**93.31**	*60.95*	**97.08**	**96.81**	**98.76**	*36.96*	**98.14**	**94.47**	*42.52*	**98.46**	**92.66**
	aECG 2	**98.14**	**91.11**	*55.53*	**93.66**	**95.44**	**98.10**	**95.66**	**95.59**	**93.58**	**98.56**	**97.23**	87.42
	aECG 3	**98.83**	*38.78*	*34.92*	*36.37*	**96.29**	*40.77*	**91.46**	**99.38**	*34.73*	**99.04**	**98.39**	**94.19**
	aECG 4	**99.45**	87.09	*63.90*	**90.41**	**97.26**	**95.63**	**95.49**	**99.84**	88.90	**99.84**	**96.76**	**99.39**
SA	aECG 1	**99.30**	**94.77**	*59.56*	**96.86**	**97.57**	**98.97**	*32.73*	**96.82**	**94.03**	*21.37*	**98.46**	**92.81**
	aECG 2	**98.91**	**92.03**	*57.52*	**93.07**	**96.20**	**97.59**	**96.05**	**96.90**	**93.00**	**97.93**	**95.15**	87.66
	aECG 3	**98.60**	*35.97*	*36.34*	*50.00*	**95.31**	*42.27*	89.51	**99.84**	*40.97*	**96.57**	**98.77**	**95.16**
	aECG 4	**99.45**	84.02	*63.64*	89.92	**98.18**	**96.06**	**96.04**	**100.00**	**90.02**	**100.00**	**98.00**	**100.00**

Significant values are in bold and italics.

**Table 3:** Results of the accuracy of R-peaks (F1) determination from extracted signals using individual tested TS-based methods on the Pregnancy dataset (using the gaus3 maternal wavelet).

TS-based method	Channel	Recording									
		r1	r2	r3	r4	r5	r6	r7	r8	r9	r10
TS	aECG 1	**93.16**	*71.19*	**96.89**	**93.85**	**97.65**	**97.92**	87.69	*79.03*	*57.45*	*17.95*
	aECG 2	**98.96**	*76.87*	*66.33*	**98.39**	**98.78**	**98.72**	**92.63**	**96.78**	87.76	*16.15*
	aECG 3	85.42	**93.09**	**97.81**	**99.47**	**97.09**	**97.24**	**97.91**	*30.63*	**95.26**	89.51
	aECG 4	*26.81*	*78.64*	*77.26*	**92.10**	**99.42**	*79.42*	81.55	86.45	*65.32*	*75.02*
TS_SVD_	aECG 1	**94.16**	*70.72*	**97.20**	**94.03**	**97.87**	**98.32**	87.26	*79.70*	*61.71*	*23.07*
	aECG 2	**99.05**	*77.85*	*67.03*	**98.74**	**99.15**	**98.68**	**92.64**	**96.54**	88.78	*17.79*
	aECG 3	85.31	**94.59**	**98.36**	**99.84**	**99.51**	**99.05**	**98.09**	*34.92*	**95.18**	**90.06**
	aECG 4	*28.35*	*79.31*	*77.15*	**92.42**	**99.60**	80.89	81.46	88.28	*70.06*	*74.60*
TS_LP_	aECG 1	**93.07**	*71.04*	**96.81**	**93.67**	**97.58**	**97.73**	87.69	*78.97*	*57.37*	*18.54*
	aECG 2	**98.76**	*76.79*	*66.26*	**98.13**	**98.64**	**98.60**	**92.54**	**96.78**	87.65	*16.15*
	aECG 3	85.30	**93.02**	**97.69**	**99.15**	**96.93**	**97.13**	**97.80**	*30.51*	**95.15**	89.39
	aECG 4	*26.95*	*78.48*	*77.28*	**91.99**	**99.31**	*79.49*	81.47	86.38	*65.32*	*74.85*
TS_SF_	aECG 1	**98.97**	*71.43*	**97.61**	**95.15**	**98.35**	**98.23**	88.41	*79.44*	*72.58*	*26.13*
	aECG 2	**99.09**	*77.23*	*64.07*	**98.61**	**99.35**	**98.56**	**92.37**	**96.11**	**91.97**	*74.90*
	aECG 3	84.94	**96.02**	**98.57**	**99.69**	**97.09**	**99.06**	**97.48**	*36.92*	**95.73**	89.19
	aECG 4	*42.95*	*79.07*	*76.83*	**92.24**	**99.76**	83.91	80.08	87.49	84.34	*74.85*
SA	aECG 1	**93.85**	*71.00*	**97.42**	**94.86**	**98.23**	**98.47**	86.02	*79.93*	*63.37*	*24.09*
	aECG 2	**99.16**	*77.58*	*65.64*	**98.92**	**99.15**	**98.66**	**92.40**	**96.76**	88.88	*18.64*
	aECG 3	86.00	**95.07**	**98.26**	**99.84**	**99.27**	**99.16**	**97.87**	*36.87*	**95.52**	89.77
	aECG 4	*29.08*	*79.77*	*76.44*	**91.85**	**99.60**	80.94	81.16	88.43	*68.20*	*74.53*

Significant values are in bold and italics.

From Tables 2 and 3, we can see that using each TS-based method, the recordings were labeled the same in most cases (high, medium, and low). When we take a closer look at the difference in accuracy between the methods for a particular channel of a recording, we can see that in most cases there is less than 5% difference between tested methods. However, in four cases the accuracy difference between tested methods was greater than 10% (Labour dataset, recording 4, channel 3; Pregnancy dataset, recording 1, channel 4; Pregnancy dataset, recording 9, channel 1, and Pregnancy dataset, recording 9, channel 4), in one case greater than 20% (Labour dataset, recording 10, channel 1) and in one case even greater than 50% (Pregnancy dataset, recording 10, channel 2). It can be seen that these were channels of recordings that achieved low accuracy for all methods. Thus, it can be concluded that there is no significant difference between the tested TS-based methods for the channels that achieved high accuracy. For the channels that achieved low accuracy, it can be hypothesized that some TS-based methods can extract the fECG signal better (at least to some degree) for these signals.

From the above Tables 2 and 3, a summary Table 4 was created, which contains for each TS-based method only the best result from each signal. The individual rows of this table contain the tested recordings, where the last row denotes the mean extraction accuracy using each method. It can be seen from the table that the SA method achieved the highest accuracy on both datasets. However, performance may vary depending on the specific recording, indicating the importance of accounting for individual differences in the fECG signal extraction process. Nevertheless, it should be noted that the results of other TS-based methods did not achieve statistically significantly lower accuracy. When mean over all recordings of both datasets simultaneously, the TS method achieved F1 = 95.71%, the TS_SVD_ method F1 = 95.93%, the TS_LP_ method F1 = 95.30%, the TS_SF_ method F1 = 95.82% and the SA method F1 = 95.99%. When we look at the difference in accuracy between the methods for individual recordings in this Table 4, we can see that it ranges from 0.4 to 3%. The largest difference was for recording 2 of the Pregnancy dataset.

**Table 4:** Highest extraction accuracies within individual recordings of both datasets for the tested TS-based methods.

Recording	Labour dataset					Pregnancy dataset				
	TS	TS_SVD_	TS_LP_	TS_SF_	SA	TS	TS_SVD_	TS_LP_	TS_SF_	SA
r1	99.45	99.45	98.99	99.45	99.45	98.96	99.05	98.76	99.09	99.16
r2	95.08	95.08	94.38	93.31	94.77	93.09	94.59	93.02	96.02	95.07
r3	60.91	61.99	60.91	63.90	63.64	97.81	98.36	97.69	98.57	98.26
r4	96.86	96.79	96.72	97.08	96.86	99.47	99.84	99.15	99.69	99.84
r5	98.33	98.33	97.04	97.26	98.18	99.42	99.60	99.31	99.76	99.60
r6	99.12	99.12	98.39	98.76	98.97	98.72	99.05	98.60	99.06	99.16
r7	95.57	96.76	94.86	95.66	96.05	97.91	98.09	97.80	97.48	97.87
r8	100.00	100.00	99.30	99.84	100.00	96.78	96.54	96.78	96.11	96.76
r9	94.98	94.18	94.47	94.47	94.03	95.26	95.18	95.15	95.73	95.52
r10	100.00	99.68	98.88	99.84	100.00	89.51	90.06	89.39	89.19	89.77
r11	98.61	98.61	97.61	98.46	98.77	–	–	–	–	–
r12	99.85	100.00	99.46	99.39	100.00	–	–	–	–	–
Mean	94.90	95.00	94.25	94.79	**95.06**	96.69	97.04	96.57	97.07	**97.10**

Significant values are in bold.

## Discussion

The quality of the input signal has a great influence on the resulting extraction. The main factor that affects the resulting signal quality is the arrangement of the electrodes and their correct mounting. Poor electrode placement results in noise that could affect the fECG signal and its resulting extraction. The success of extraction may also be affected by the gestation age and its position in the pregnant woman’s abdomen, as the fHR changes during development. Recordings that contain aECG signals with substandard quality produced low fQRS complex detection accuracy. This was due to the fact that the level of the fetal component was very low compared to the maternal component and in some cases even invisible. Some signals also suffered from noise. For these signals, effective extraction is almost impossible, and therefore it is important to pay close attention to the correct positioning of the sensing electrodes and the setup of the measurement system when acquiring them. Table 5 shows for each recording of both datasets which input aECG signals have low, medium and high quality. This table is intended to help the future authors in selecting input signals for the extraction methods and can also serve as a check for automatic classifiers of input aECG signals based, for example, on evaluation using input signal quality index (SQI) parameters. This table was created based on the results from Tables 2 and 3, where the criteria were determined as follows:

- Signals with low-quality: F1 = 0–80%.
- Signals with medium-quality: F1 = 80–90%.
- Signals with high-quality: F1 = 90–100%.

**Table 5:** Determination of the quality of input aECG signals for subsequent fECG signal extraction.

Recording	Labour dataset			Pregnancy dataset		
	High quality channels	Medium quality channels	Low quality channels	High quality channels	Medium quality channels	Low quality channels
r1	1, 2, 3, 4	–	–	1, 2	3	4
r2	1, 2	4	3	3	–	1, 2, 4
r3	–	–	1, 2, 3, 4	1, 3	–	2, 4
r4	1, 2	4	3	1, 2, 3, 4	–	–
r5	1, 2, 3, 4	–	–	1, 2, 3, 4	–	–
r6	1, 2, 4	–	3	1, 2, 3	4	–
r7	2, 4	3	1	2, 3	1, 4	–
r8	1, 2, 3, 4	–	–	2	4	1, 3
r9	1, 2	4	3	3	2	1, 4
r10	2, 3, 4	–	1	–	3	1, 2, 4
r11	1, 2, 3, 4	–	–			
r12	1, 3, 4	2	–			

**Figure 4:**
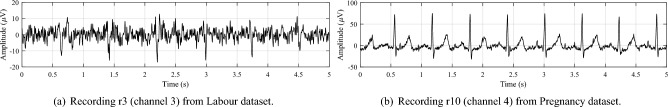
Example of low quality aECG input signals leading to insufficient fECG signal extraction.

The biggest problem with signals marked as low quality was frequency noise, which was present in the signal despite filtering with a band-pass FIR filter with cutoff frequencies of 5–70 Hz (see Fig. 4a). The data used were also filtered by the dataset authors themselves as mentioned in the section describing the datasets used. However, in the signals it was at least possible to see that the removal of powerline interference was effectively done. Furthermore, there was no isolinear fluctuation of the signals because even these low frequencies were effectively removed by the authors of the datasets.

Unfortunately, the detection of low efficiency, for some of the signals we identified as low quality, was due to the measurement of aECG signals with no visible fECG signal (see Fig. 4b). As already mentioned, this could have been a problem with the fetal position. In particular, we would talk about signals where only one or two signals were marked as low quality and the others as medium quality or high quality. However, for recording r3 from the Labour dataset and r10 from the Pregnancy dataset, most of the signals were marked as low quality. Here, all signals and therefore the whole of both recordings were under-measured, which could be due to different reasons.

Furthermore, it was very interesting to analyze the effect of applying the TS-based method on the amplitude of fetal R-peaks. In fact, TS-based methods have the additional problem that fetal R-peaks can be partially removed during maternal subtraction. Therefore, the amplitudes of fetal R-peaks were determined for all input aECG signals of both datasets using reference annotations. Subsequently, amplitude averaging was performed for each input aECG signal. The same was done for the extracted signals, again using the reference annotations to eliminate the effect of minor inaccuracies of the CWT detector used. For this analysis, we used the extracted fECG signals using the SA method because it achieved the best result according to our study. The mean change in amplitude of the fetal R-peaks can then be seen for the Labour dataset in Table 6 and for the Pregnancy dataset in Table 7. The tables show that the assumed amplitude change is present in the extracted signals. In most cases, it was a change in amplitude of a few microV. However, for some signals, and especially from the Pregnancy dataset, it can be seen that in some cases it was a change in amplitude of practically half.

**Table 6:** Mean change in fetal R-peak amplitude in extracted fECG signals relative to input aECG signals (labour dataset).

Recording	fQRS ($\mu$V)											
	Channel 1			Channel 2			Channel 3			Channel 4		
	aECG	fECG	Difference	aECG	fECG	Difference	aECG	fECG	Difference	aECG	fECG	Difference
r1	17.35	15.38	1.96	19.94	18.12	1.81	17.51	16.27	1.24	28.07	26.16	1.92
r2	37.65	30.66	6.99	28.77	24.25	4.52	9.97	8.89	1.08	19.01	16.25	2.76
r3	11.31	9.35	1.96	11.45	10.01	1.44	6.47	5.65	0.81	11.37	9.73	1.64
r4	27.24	21.56	5.69	21.76	18.47	3.29	11.16	9.74	1.42	13.68	11.62	2.06
r5	17.67	15.67	1.99	21.74	19.84	1.91	17.33	16.56	0.77	29.08	27.39	1.69
r6	32.85	26.32	6.53	27.18	22.68	4.50	9.82	8.78	1.05	18.60	15.83	2.77
r7	6.73	5.16	1.58	16.41	13.84	2.58	10.74	9.81	0.94	16.87	15.02	1.85
r8	18.85	16.95	1.91	21.35	20.12	1.22	19.08	18.25	0.83	31.11	29.51	1.60
r9	33.46	26.84	6.62	28.52	23.47	5.05	13.46	11.61	1.86	19.37	16.32	3.05
r10	6.99	5.09	1.90	16.55	14.07	2.48	12.00	10.70	1.30	19.60	18.16	1.44
r11	20.72	18.31	2.40	25.77	24.81	0.96	20.40	19.27	1.13	33.17	31.29	1.88
r12	19.08	16.77	2.31	22.70	20.04	2.66	19.18	17.90	1.28	29.67	28.38	1.29

**Table 7:** Mean change in fetal R-peak amplitude in extracted fECG signals relative to input aECG signals (Pregnancy dataset).

Recording	fQRS ($\mu$V)											
	Channel 1			Channel 2			Channel 3			Channel 4		
	aECG	fECG	Difference	aECG	fECG	Difference	aECG	fECG	Difference	aECG	fECG	Difference
r1	12.02	10.83	1.19	11.23	10.78	0.45	11.84	10.46	1.38	11.67	6.48	5.19
r2	15.98	10.41	5.57	16.91	11.44	5.47	19.99	13.92	6.08	14.28	11.04	3.25
r3	13.62	10.73	2.89	9.28	7.25	2.03	14.98	11.41	3.57	8.96	7.87	1.10
r4	14.92	11.71	3.21	17.07	14.04	3.03	20.17	16.08	4.09	9.79	7.67	2.12
r5	16.40	12.27	4.13	17.36	14.72	2.63	27.06	24.91	2.15	18.75	17.45	1.30
r6	8.00	6.04	1.96	8.11	6.12	1.99	11.27	8.67	2.60	8.17	5.80	2.37
r7	7.75	6.62	1.13	9.11	7.82	1.29	10.50	8.89	1.61	10.30	6.22	4.08
r8	8.34	5.18	3.16	5.53	3.95	1.58	7.24	4.84	2.40	8.57	4.71	3.86
r9	12.99	5.55	7.44	10.08	5.93	4.15	11.84	8.51	3.33	8.30	6.49	1.81
r10	9.10	4.29	4.81	7.37	4.25	3.13	13.25	10.67	2.58	10.65	9.00	1.65

The following Table 8 compares the results of the proposed method with studies dealing with single-channel fECG signal processing. It is obvious from this table that it is very difficult to accurately compare the results obtained in the study. This is because different studies use different evaluation parameters, datasets or even approaches to fECG single extraction. The results obtained in our study were in most cases higher (or comparable) than other studies focusing on single-channel signal processing methods. For comparisons, studies that used at least one of the ACC, SE, PPV and F1 evaluation parameter were mainly selected. Higher accuracy was achieved in two of the studies mentioned^38,39^. However, upon closer examination of the experiment performed by the authors of these studies, it can be seen that they only used signals from the tested datasets that provides good results. For example, for a similar dataset ADFECGDB, which is basically an older version of the Labour dataset, authors selected only the input aECG channels from 5 recordings that have high quality signals. These signals have high quality also according to Table 5 in this study. It is therefore clear that in this study the resulting accuracy is reduced due to the results from the lower quality recordings.

**Table 8:** Results comparison with studies focused on singlechannel fECG extraction.

Authors	Algorithm	Dataset	Results
Sarafan et al.^17^	TS	Physionet Challenge 2013	With/without motion noise F1 = 71.02/82.65%
Kanjilal et al.^29^	TS_SVD_	Composite mECG signal 1 pregnant woman	–
Vullings et al.^30^	TS_LP_	49 synthetic aECG recordings 7 real aECG recordings	SE = 90.1%, PPV = 94.2%
Cerutti et al.^31^	TS_SF_	20 pregnant women	–
Martens et al.^32^	SA	20 pregnant women	ACC = 85%
Andreotti et al.^33^	TS_SVD_, TS_SF_, TS_EKF_	145.8 h of multichannel syntetic signals	F1 = 96%
Souriau et al.^12^	DTW	FECGSYNDB Pregnancy dataset	–
Liu et al.^35^	TM	Physionet Challenge 2013	F1 = 95%
Gurve et al.^37^	NMF	ADFECGDb Physionet Challenge 2013	SE = 95.3%, PPV = 94.6%, F1 = 94.8% F1 = 84%
Krupa et al.^38^	FrFT-DWT	Daisy dataset Physionet Challenge 2013 Real-time signals acquired using Powerlab	ACC = 98.12%, SE = 98.85%, PPV = 99.16%, F1 = 99.42%
Krupa et al.^39^	ST	Daisy dataset Physionet Challenge 2013 ADFECGDB NIFEA DB	ACC = 96.6%, SE = 96.6%, PPV = 100%, F1 = 98.27% ACC = 97.37%, SE = 98.61%, PPV = 98.72%, F1 = 98.67% ACC = 98.55%, SE = 99.16%, PPV = 99.38%, F1 = 99.27% ACC = 99.87%, SE = 99.94%, PPV = 99.93%, F1 = 99.94%
Proposed	TS TS_SVD_ TS_LP_ TS_SF_ SA	Labour dataset Pregnancy dataset	F1 = 95.71% F1 = 95.93% F1 = 95.30% F1 = 95.82% F1 = 95.99%

**Figure 5:**
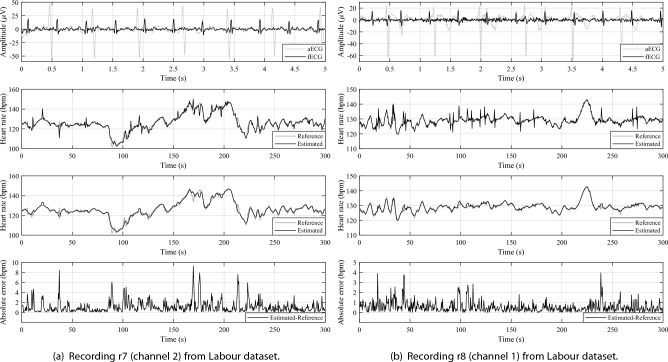
Demonstration of fECG signal extraction using SA method and subsequent fHR estimation.

Figure 5 shows examples (first 5 s of signals) of successful extractions of fECG signals using the SA method. For both subfigures there are always samples of the input aECG signal (grey waveform) and the subsequent extracted fECG signal (black waveform) in the upper graph. The second graphs from the top shows the calculated fHR from the reference annotations (grey plot) and using the detected R-peaks in the extracted fECG signal (black plot). In these plots, slight deviations (peaks) from the reference can be seen in the estimated fHR, which is due to a slight shift of the detected R-peaks. In the following graphs, a moving averaging with a window length of 5 samples is applied to the estimated fHR, which removes the mentioned peaks and preserves the trend of the fHR with respect to the reference. In the last graphs, a subtraction of the estimated fHR from the reference fHR is performed to show the error signals. These error signals can be seen to have a small amplitude and hence just a small deviation from the reference.

The results of this study could be higher if some signal smoothing method is used as a final step^55^ or if an optimization technique is used^56^. However, in this study, the focus was primarily and only on the comparison of various different TS-based methods in fECG signal extraction. The result of this study can be used in the design of an efficient hybrid system in which the SA method would be used as the main part of the extraction system.

Future research will focus on testing new signal processing methods that can be used as a sub-part of a hybrid system. Along with testing methods, the aim will be to test new optimisation algorithms, especially those inspired by nature. Much attention will also be paid to testing single-channel signal processing methods. However, a major problem is the selection of input signals, so the simultaneous research goal focuses on SQI testing. The goal is to develop a system that evaluates whether the input aECG signal is suitable for extraction, contains a enoungh visible fetal signal and does not have too much noise. This problem has been addressed by many authors, but no system exists (that achieve accuracy approaching almost 100%) yet that automatically evaluates input aECG signals. Promising results were achieved in the study^57^, where they used a supervised machine learning approach for automatic selection. Their results were very interesting when they performed an experiment on 10336 5-second signal segments obtained from a real data set of multi-channel transabdominal recordings obtained from 55 volunteer pregnant women between 21 and 27 weeks of pregnancy. They achieved an accuracy of over 86% and more than 88% of the channels marked as informative were correctly identified.

Next, attention will be paid to the multichannel determination of fHR. That is, when performing single-channel fECG signal extraction on multiple input signals, multiple fECG signals are received. The goal will be to separately detect R kmits from these signals and then compare them with each other using different methods to achieve more accurate fHR estimation. Alternatively, the goal will be to adjust the fHR or multiple detected fHR curves from the extracted fECG signals. In summary, the goal will be to achieve the most accurate estimate of fHR relative to the reference when multiple extracted fECG signals can be used. Another aim of the research will be to perform morphological analysis, i.e. analysis of ST segment, QT interval length, etc. The mentioned segments and lengths are very important sources of information about the health status. A major advantage of TS-based methods is that they do not interfere with the morphology of the extracted fECG signal. In fact, a large number of classical signal processing methods such as WT or EMD have the problem of morphology corruption. This means that TS-based methods can be considered as suitable in terms of the possibility to perform ST analysis.

## Conclusion

This study dealt with fECG signal processing using TS-based methods. For experiments, two datasets containing real signals including annotations were used: Labour dataset and Pregnancy dataset. The aim of the study was to compare several methods (TS, TS_SVD_, TS_LP_, TS_SF_ and SA) with each other and to determine the accuracy achieved on the individual signals of the datasets used. In addition, many types of maternal wavelets used for the CWT detector were tested to see what effect this has on the detection accuracy. From the testing it was evident that the best performance was achieved using the Gaussian family of wavelets and the best result was achieved using the gaus3 maternal wavelet. The accuracy of the selected methods was evaluated by determining the statistical parameter F1. The highest mean extraction accuracy on the two datasets used was achieved using the SA method (F1 = 95.99%). In addition, the quality/usability of the input signals of the individual recordings of the datasets used in this work was determined. This work supports the claim that TS-based methods are suitable for fECG extraction. Based on their effectiveness, these methods could be used in the future as part of hybrid systems. Combined these methods with another signal processing method and taking its advantages, even higher fECG signal extraction accuracy could be achieved (Supplementary Informations S1 and S2).

### Supplementary Information

Supplementary Information.

Supplementary Information.

## Data Availability

The datasets analysed during the current study are available on a server at the generalist repositories (figshare) integrated with Scientific Data Journal. The commission approval number is: NN-013-345/02 and can be found at https://doi.org/10.6084/m9.figshare.c.4740794^43^.
